# We Want to Go, but There Are No Options: Facilitators and Barriers of Transportation Among Diverse Older Adults

**DOI:** 10.1093/geroni/igaa057.2464

**Published:** 2020-12-16

**Authors:** Quichang Cao, Arati Maleku, Katie White, Basar Ozbilen, Holly Dabelko-Schoeny

**Affiliations:** 1 The Ohio State University, Columbus, Ohio, United States; 2 Age Friendly Columbus, Columbus, Ohio, United States

## Abstract

Transportation plays an important role in social connectedness and quality of life among older adults. Despite the increasing number of diverse older adults in the U.S., few studies have explored the barriers and facilitators of transportation among this group. We conducted eight 90-minute focus groups in six languages (English, Nepali, Khmer, Somali, Russian and Mandarin) with older volunteers (N=70) in a large Midwestern metropolitan city. Using the Rapid and Rigorous Qualitative Data Analysis (RADaR) technique, four transportation determinants emerged: (1) Service: affordability, accessibility, availability, and reliability, lack of options, and service coordination (2) Built environment: safety and walkability; (3) Social environment: language barriers and lack of information, neighborhood cohesion; and (4) Individual determinants: ability to drive, walk, and family support system. Results reveal the interconnectedness of multi-level determinants and the need for a systematic approach to improve transportation access among diverse older adults.

